# Enrichment of ODMR-active nitrogen-vacancy centres in five-nanometre-sized detonation-synthesized nanodiamonds: Nanoprobes for temperature, angle and position

**DOI:** 10.1038/s41598-018-23635-5

**Published:** 2018-04-03

**Authors:** Shingo Sotoma, Daiki Terada, Takuya F. Segawa, Ryuji Igarashi, Yoshie Harada, Masahiro Shirakawa

**Affiliations:** 1 Department of Molecular Engineering, Graduate School of Engineering, Kyoto University, Nishikyo-Ku, Kyoto, 615–8510 Japan; 2 Laboratory for Solid State Physics, Eidgenössische Technische Hochschule (ETH) Zürich, CH-8093, Zürich, Switzerland; 3 PRESTO, Japan Science and Technology Agency, Kawaguchi, 332–0012 Japan; 4 Institute for Protein Research, Osaka University, Yamadaoka, Suita, Osaka, 565–0871 Japan; 5 Institute for Integrated Cell-Material Sciences (WPI-iCeMS), Kyoto University, Yoshida-Honmachi, Sakyo-ku, Kyoto, 606–8501 Japan

## Abstract

The development of sensors to estimate physical properties, and their temporal and spatial variation, has been a central driving force in scientific breakthroughs. In recent years, nanosensors based on quantum measurements, such as nitrogen-vacancy centres (NVCs) in nanodiamonds, have been attracting much attention as ultrastable, sensitive, accurate and versatile physical sensors for quantitative cellular measurements. However, the nanodiamonds currently available for use as sensors have diameters of several tens of nanometres, much larger than the usual size of a protein. Therefore, their actual applications remain limited. Here we show that NVCs in an aggregation of 5-nm-sized detonation-synthesized nanodiamond treated by Krüger’s surface reduction (termed DND-OH) retains the same characteristics as observed in larger diamonds. We show that the negative charge at the NVC are stabilized, have a relatively long T_2_ spin relaxation time of up to 4 μs, and are applicable to thermosensing, one-degree orientation determination and nanometric super-resolution imaging. Our results clearly demonstrate the significant potential of DND-OH as a physical sensor. Thus, DND-OH will raise new possibilities for spatiotemporal monitoring of live cells and dynamic biomolecules in individual cells at single-molecule resolution.

## Introduction

Nitrogen-vacancy centres (NVC) in diamond are a well-studied type of fluorescent chromophore centres, comprising a substitutional nitrogen atom and an adjacent vacancy in the crystal lattice. In recent years, NVC-containing nanodiamonds have been attracting increasing attention as imaging reagents and biophysical sensing probes, particularly in the research field of biological imaging^1–3^, because of their low toxicity^4,5^, chemical and biological modifiability^6,7^, and extraordinary photostability^8^. Moreover, an NVC can possess a pair of electrons in the triplet state, whose spin-energy levels are sensitive to changes in a variety of surrounding physical conditions, such as electric^9,10^ and magnetic^11,12^ fields and temperature^13^, and whose spin states are strictly coupled to fluorescence photoemission at room temperatures under ordinary pressures^8^. Therefore, NVC-containing nanodiamonds are one of the most promising optical cellular imaging probes and can be used for intracellular physical quantitation based on optically detected magnetic resonance (ODMR) measurements^1,3^.

Despite this tremendous potential, however, the biological applications of nanodiamonds in actual research remain limited to demonstration experiments, mainly because of their size. Everyday biological research commonly makes use of fluorescence reagents, including fluorescent proteins and dyes that are comparable in size to ordinary intracellular biomacromolecules as small as 5 nm. In contrast, the ordinarily available NVC-containing nanodiamonds produced by high-pressure high-temperature, chemical vapour deposition or other methods are not smaller than 20-nm diameter^1,3,11^. For this reason, novel approaches are eagerly anticipated to produce much smaller NVC-containing nanodiamonds that have stable ODMR-active negatively charged NVCs.

Detonation-synthesized nanodiamonds (DNDs) represent some of the smallest diamond crystallites and are produced as colloidal dispersions as small as 5 nm with a uniform size distribution^14,15^. Recent studies have reported that DNDs can host NVCs in their crystal structure. Indeed, ODMR measurements based on NVCs in DNDs have been reported in several studies^16–18^. Moreover, stable photon emission and high-contrast ODMR measurements have also been performed using nanodiamonds smaller than 5 nm^19^. Those results imply that the potential exists to stabilise and enrich the NVCs in DNDs. However, DNDs have not yet been used in sensing applications because of the probability of finding an ODMR-active NVC in a DND is low. In 5-nm DNDs, NVCs are spatially very close to the particle surface, where terminal groups can strongly affect the charge state of the NVC, destabilize the negative charge on the defect and thereby decrease the ODMR signals^20^.

Here we describe stability enhancement of the ODMR-active negatively charged NVCs in a DND surface-terminated with hydroxyl (OH) groups by Krüger’s borane reduction^21^, and demonstrate three different types of ODMR-based measurement using these DNDs. To obtain strong ODMR signals using DNDs, we focused on charge-state conversion of NVCs using surface oxygen terminations^22–24^, which increased the population of negatively charged NVCs and stabilized their triplet electrons in OH-terminated DND (DND-OH). We found that the NVCs in DND-OH provide adequate ODMR signals, have relatively long spin relaxation times of of up to 4 μs, and can be successfully applied to ODMR-based thermosensing, one-degree orientation determination and nanometre-resolution imaging. All experiments were performed on individual NVCs in an aggregation of OH-terminated DNDs.

## Results

**Figure 1:**
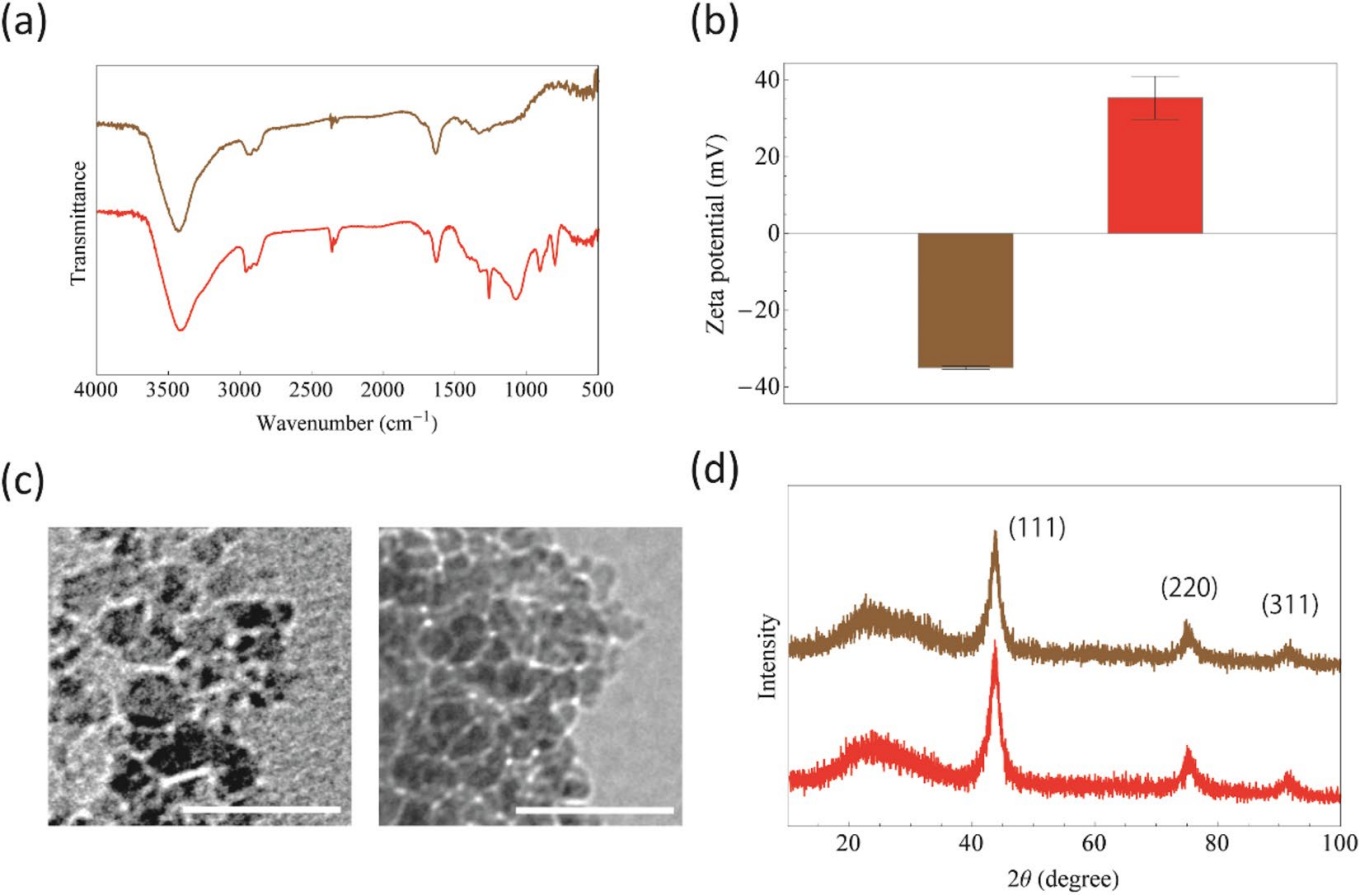
Characterization of DND-bare and DND-OH. (**a**) Fourier transform infrared spectroscopy spectra of DND-bare (brown) and DND-OH (red). (**b**) Zeta potentials of DND-bare (brown; −35.0 ± 0.5 mV) and DND-OH (red; 35.4 ± 5.6 mV). (**c**) Transmission electron microscope images (scale bar 30 nm) of DND-bare (left) and DND-OH (right). (**d**) X-ray diffraction spectra of DND-bare (brown) and DND-OH (red). Three peaks corresponding to the (111), (220) and (311) diffraction lines of diamond were observed.

NVCs exist in one of two different charge states, a neutral state (NV^0^) and a negatively charged state (NV^−^), which are spectroscopically characterized by zero-phonon lines at 575 nm and 638 nm, respectively, and broad and intense phonon sidebands at room temperature^25,26^. Among the two charge states, only NV^−^ has stable triplet electrons, which can provide physical information on the environment around the NVC by means of ODMR measurements. Here, to stabilize negatively charged NVCs in DNDs, we chemically treated the surface terminal groups by borane reduction^21^ to obtain DND-OHs (Fig. 1). After this step, the terminal functional groups on the surface of the DNDs seemed to be efficiently exchanged by OH groups (Fig. 1a); the δ-OH vibrations around 1250 cm^−1^ and C-O vibrations around 1000–1200 cm^−1^ in alcohol groups are increased in the spectrum of DND-OHs^27^. In addition, an increase in the zeta potential from −35.0 ± 0.5 mV for non-treated DNDs (“DND-bare”) to 35.4 ± 5.6 mV for DND-OHs supports the successful surface modification (Fig. 1b). To confirm that the particle size and diamond crystal structure of DNDs were not altered by the thermal and chemical treatments, we observed the DND-OHs by transmission electron microscope (TEM) and X-ray diffraction (XRD), which showed that the size and structure were retained before and after these treatments (Fig. 1c and d). Hence, DND-OHs were successfully obtained by borane reduction of DNDs.

**Figure 2:**
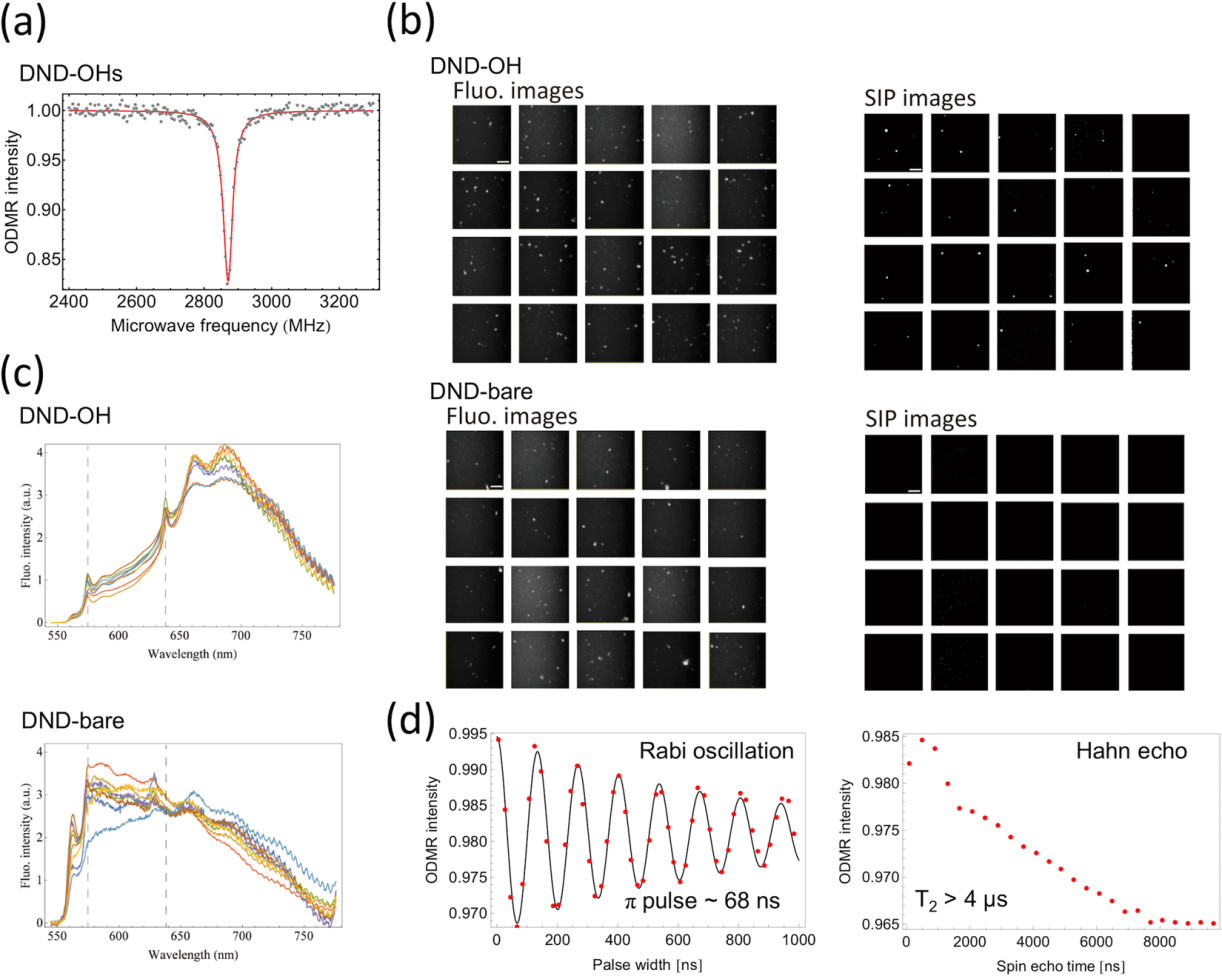
Fluorescence and ODMR properties of DND-bare and DND-OH. (**a**) Typical ODMR spectra of clusters of DND-OH. (**b**) Typical fluorescence (left) and NV^−^selective imaging protocol (SIP) derived (right) images of DND-OH (upper) and DND-bare (lower), which were dispersed in water, sonicated and strewn on a glass plate (scale bars 10 μm). (**c**) Fluorescence spectra of DND-OH (upper) and DND-bare (lower) acquired from randomly selected DND clusters (n = 8). Broken lines at 575 and 637 nm represent the zero-phonon lines of NV^0^ and NV^−^, respectively. (**d**) Rabi oscillation (left) and *T*_2_ relaxation time (right) of a typical NV^−^ in DND-OH. *T*_2_ was measured by a simple Hahn echo sequence using a single π pulse, and evaluated by its 1/e-time. Both pulse ODMR measurements were performed with an applied magnetic field at the resonance of 2785 GHz (see Fig. 2(a) lower). Both ODMR spectra in Fig. 2(a) and both pulse ODMR experiments in Fig. 2(d) were recorded on a confocal setup.

Next, we determined whether surface termination with OH groups effectively converted NV^0^s to NV^−^s and enhanced the ODMR signals of DNDs (Fig. 2). First, DNDs were aggregated into granules of reliably observable size (Fig. S1) and applied to a glass coverslip; we then swept the microwave frequency from 2670 to 3070 MHz while monitoring fluorescence intensity, whereby a decrease in intensity indicated resonant spin excitations of the triplet-electron spins at an NV^−^. As result, DND-OHs showed a fluorescence decrease centred around 2870 MHz, corresponding to the zero-field splitting frequency of the spin-triplet ground state (Fig. 2a, upper panel).

For a direct quantitative comparison of non-treated DND-bare with DND-OH, DNDs were observed by the ODMR-based microscopic imaging technique using a selective imaging protocol (SIP)^2^ at 2870 MHz at zero magnetic field, which specifically filters ODMR-active NVCs with a high contrast (Fig. 2b). For DND-OH, out of 309 fluorescent points containing NV^0^ and NV^−^, we observed 51 fluorescent points with a clear signature from NV^−^. For DND-bare, however, no fluorescent points attributed to NV^−^ could be seen during the selective imaging. This indicates that 17% of ODMR-inactive fluorescent DND spots were changed into ODMR-active ones after the OH termination step. Please note that we did not evaluate single DNDs but DND clusters in this experiment. The fluorescence emission spectra of DND-OH and DND-bare confirmed that the increased ODMR activity stems from the conversion of NV^0^ to NV^−^ (Fig. 2c). As a result, we consider that the negative charge state of NV^−^ in DNDs was stabilized by the presence of surface OH groups.

This procedure enabled us to easily identify ODMR-active single NVCs in DND-OHs applied at lower concentration, and to perform various ODMR measurements as follows. We note that the observed values for the *T*_2_ spin relaxation time of the NVC in DND-OH were about 1–4 μs (Fig. 2d), which is the same as or better than reported previously^19^. This indicated that DND-OH might have the potential for use in complex ODMR experiments requiring relatively long pulse sequences.

**Figure 3:**
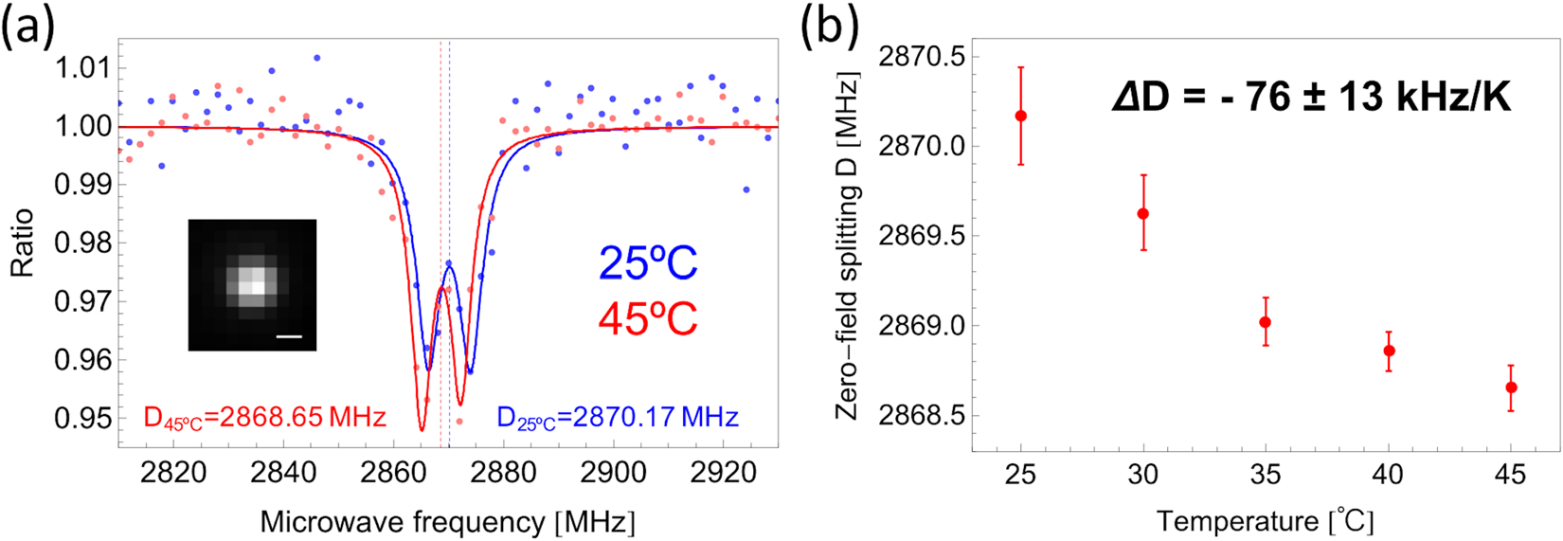
Typical temperature dependence of DND-OH. (**a**) Temperature-dependent change in ODMR spectra recorded at 25 and 45 °C with acquisition times of 10 min. The raw data obtained, best-fit curve, and zero-field splitting frequency *D* are represented by dots, solid line and broken line, respectively. The best-fit model was based on equation (1). D_25 °C_ and D_45 °C_ are the *D* values estimated from the best-fit curves at 25 and 45 °C, respectively. Inset shows the measured fluorescent bright spot of DND-OH (scale bar 500 nm). (**b**) Temperature dependence of *D*, *ΔD*. Spectra were recorded at 25, 30, 35, 40 and 45 °C by using the same bright spot as in (**a**). Red dots represent estimated *D* values. Error bars were calculated on the basis of fitting errors between the model curves and the observed data. Broken gray line represents the linear approximation with a slope of *ΔD*.

To evaluate the performance of DND-OH in ODMR measurements, we carried out thermosensing^13^, orientation determination^1^ and super-resolution imaging^28^ by different ODMR-based measurement techniques as follows. A pair of triplet electron spins at an NVC is described by the following spin Hamiltonian:

1 $$H=g{\mu }_{B}BS+D[{S}_{Z}^{2}-\frac{1}{3}S(S+1)]+E({S}_{x}^{2}-{S}_{y}^{2}),$$

where *g*, *μ*_*B*,_ *B*, *S*, *D* and *E* are the *g* factor for an electron (*g* = 2.0), Bohr magneton, external magnetic field, electron spin vector (*S* = 1 for triplet state), fine-structure zero-field splitting and strain-induced splitting coefficient, respectively. For instance, it has been previously reported that *D* changes with temperature (owing to thermally induced lattice strains^13^) approximately linearly at a rate of about −80 to −70 kHz/K within a physiologically significant temperature range^13^. We therefore determined whether the DND-OHs retained the same temperature dependence of the spin energy state (Fig. 3). As a result, we observed low-frequency shifts of ODMR spectra with increasing temperature (Fig. 3a). The temperature dependence of the zero-field splitting frequency *D* was estimated to be *ΔD* = −76 ± 13 kHz/K (Fig. 3b), which is similar to the value determined previously using bulk diamonds and nanodiamonds with diameters of several tens of nanometres^3,13^. An NVC-containing nanodiamond has been reported to act as a reliable nanoscale thermometer that can be used for intracellular thermometry^3^, and has significant potential to end the controversy of whether spatial temperature variations are caused by intracellular thermogenesis, particularly in mitochondria^29–31^. A DND-OH is much smaller than mitochondria (~1–2 μm), and is therefore likely to be useful for this kind of measurement, which requires such high spatial resolution.

**Figure 4:**
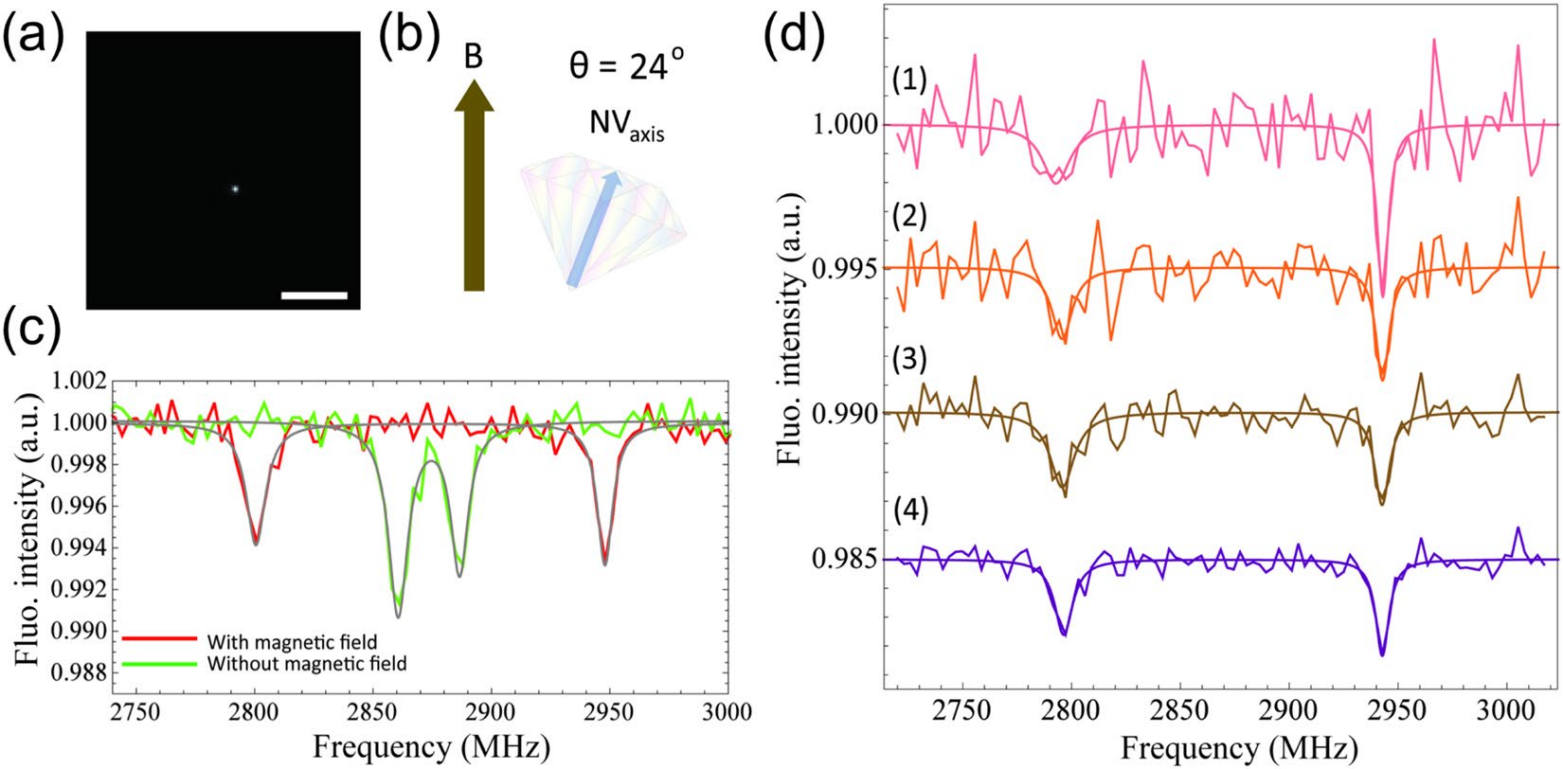
Determination of the N–V orientation. (**a**) Fluorescence image (scale bar 10 μm), (**b**) schematic of the relative angle of the N–V axis to the applied magnetic field and (**c**) corresponding ODMR spectra. Red and green plots in (**c**) represent ODMR spectra with and without a 28 Gauss external magnetic field. With the field applied, Zeeman splitting is observed between the two peaks at 2801 and 2948 MHz. (**d**) ODMR spectra with acquisition times of (1) 5 s, (2) 10 s, (3) 20 s and (4) 50 s. Spectra have been fitted with two Lorentzian functions.

Next, we determined the N–V orientation in the DND-OHs (Fig. 4a); due to the Zeeman effect, this orientation is known to affect the resonant frequency of the NVC in an external magnetic field according to the first term, *gμ*_*B*_*BS*, in equation (1), and is a practically important parameter in intracellular nanometric dynamics, as previously reported^1^. The angle *θ* between the axis of N–V and the known external magnetic field *B* in DND-OH was uniquely determined (Fig. 4b; see also Fig. S2) by the best fit of the ODMR spectrum (Fig. 4c, green and red solid lines) to equation (1) (Fig. 4c, grey solid lines). In this orientation determination, an angular accuracy of ±0.3° was achieved in an acquisition time of 50 s (Fig. 4d, plot 4), where the accuracy was estimated by a Monte Carlo method (see Fig. S3 for details). Moreover, the average errors in acquisition times of 5, 10, 20, and 50 s were estimated as ±4.0°, ±1.5°, ±0.5° and ±0.3°, respectively (Fig. 4d). Note that the number of peaks and the intensity of the ODMR signals did not change when the external magnetic field was applied. This strongly suggests that the orientation of a single NVC in a crystal was measured. As previously mentioned by McGuinness *et al*.^1^, using this method with 5-nm DND-OH would allow real-time tracking of molecular structural dynamics over an order-of-magnitude longer duration than commonly used measurement methods.

**Figure 5:**
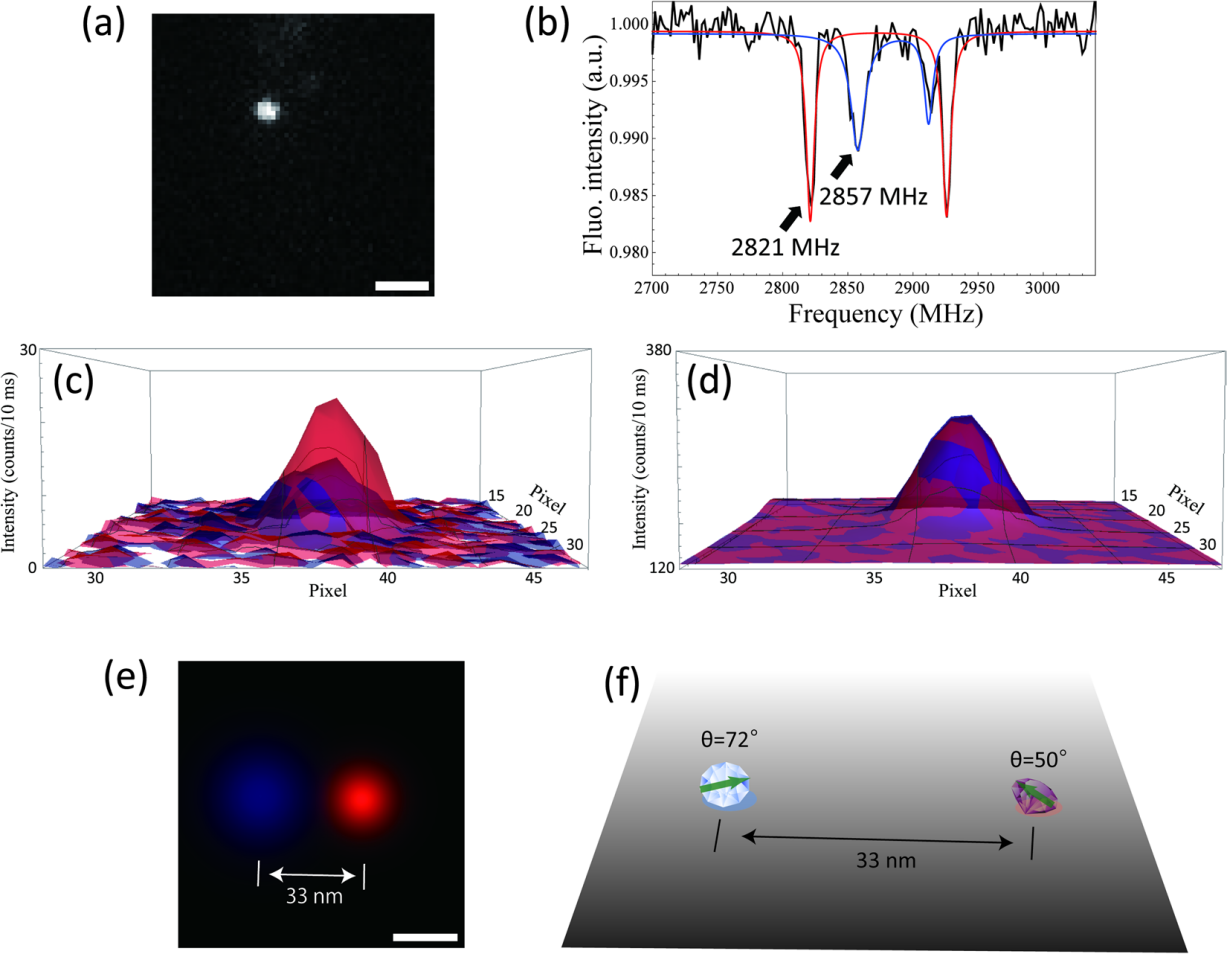
Super-resolution imaging experiments based on ODMR using DND-OHs. (**a**) Fluorescence image of DND-OHs (scale bar: 1 μm). (**b**) ODMR spectrum of the DND-OHs with fitting of the two NV-centers (red and blue) in (**a**). Two individual ODMR signals are observed from the DND-OH. (**c**) 2D NVC-selective images with microwave irradiation at 2821 (red) and 2857 MHz (blue) and (**d**) fluorescence images obtained without microwave irradiation. (**e**) Reconstructed super-resolution image of (**c**) fitted by a 2D Gaussian (scale bar 20 nm). (**f**) Schematic of the simultaneous measurement of super-resolution imaging and relative orientation measurement.

Lastly, we performed super-resolution imaging experiments based on an ODMR technique using DND-OHs (Fig. 5). It is well known that the centre position of an isolated single fluorescent probe can be determined within 1-nm accuracy as the centre of a 2D-Gaussian function fitted to the fluorescence image^32^. This concept has been extended to localization-based super-resolution imaging approaches, such as PALM^33^ and STORM^34^, by coupling it with stochastic excitation or selective detection of fluorophores. Therefore, we coupled it with ODMR-based selective detection at a known external magnetic field^2^ (see also Fig. S4) and performed super-resolution imaging^28^. To distinguish two NVCs in a DND-OH within the diffraction limit (Fig. 5a), we obtained the overall ODMR spectrum given by both of them (Fig. 5b). This spectrum comprised two different spectra (red and blue plots), arising from the two individual NVCs. Therefore, by using microwaves at 2821 and 2857 MHz, we constructed an individual selective image of each NVC (Fig. 5c, red and blue, respectively), and successfully distinguished the positions of the two NVCs. By contrast, these two NVCs could not be distinguished by using only fluorescence information (Fig. 5d, red and blue). Moreover, by fitting each of NVC-selective image with a 2D-Gaussian function, we determined the centre position of the NVCs and estimated the position-determination errors due to shot noise^35^ and instrumental and environmental instability (Fig. S5). Thus, the distance was as small as 33 nm and the errors were estimated as 11 and 7 nm (Fig. 5e, red and blue, respectively). Note that such a fine determination of positions is virtually meaningless with the commonly used nanodiamonds of several tens of nanometres. Therefore, 5-nm DND-OHs have a strong potential for applications of ODMR super-resolution imaging. These orientations of the two NVCs to the external magnetic field were determined as 50° and 72°, respectively (Fig. 5f, red and blue). Thus, we can simultaneously determine the N–V orientations while recording super-resolution images. In other words, all of the three-dimensional six degrees-of-freedom motion (3D translations and 3D rotations) of a nanometric rigid body, such as a biomolecule, can be determined by using DND-OHs.

## Discussion

Our experiments show that an NVC in a Krüger’s DND-OH carries a stable negative charge and possesses stable NV^−^, which retain the similar characteristics as those of NVCs in diamond particles of several tens of nanometres. NVCs in DND-OH have a *T*_2_ relaxation time of about 1–4 μs. To the best of our knowledge, this is the longest reported coherence time (Hahn echo) for NVCs in such small nanodiamonds. It will be interesting to apply dynamical decoupling schemes on DND-OHs to see how much the coherence times could be further prolonged^36^. We also show for the first time that various ODMR-based measurements such as thermosensing, orientation determination and super-resolution imaging can be carried out with 5nm-sized DND-OHs containing a single NVC. In this way, we have clearly demonstrated the utility of DND-OHs for measurements based on basic and robust frequency-sweep ODMR techniques using continuous microwaves. In recent years, many faster, highly sensitive, and more powerful pulse ODMR techniques have been reported for a variety of purposes. Such techniques enable us, for instance, to measure temperature with sub-Kelvin accuracy on the millisecond timescale^3,37^, a temporal resolution that is generally suitable for real-time observation of live cells. However, the particle size of the commonly used large nanodiamonds is not always small enough either to determine where biological events occur or to observe how biomolecules behave within a cell. By contrast, a DND-OH particle is as small as a molecule of green fluorescent protein, the most frequently used material for labelling biomolecules in live-cell imaging. Via its attachment to a biomolecule, therefore, a DND-OH is likely to be applicable to the detection of a wide range of intracellular biological events caused by biomolecular function. In addition, we can efficiently perform molecular measurements based on Förster resonance energy transfer (FRET)^38^, a process in which the proximity between the donor and the acceptor (typically in the 1–10 nm range) is essential for efficiency, by using DND-OH as an extremely photostable FRET acceptor. Fortunately, an H3 centre (or N–V–N centre) in a diamond crystal lattice emits stable green fluorescence with a wavelength range that overlaps extensively with the fluorescence excitation spectrum of NVCs^39^, which therefore acts as a potential donor in photostable FRET. Our study thus provides a means of monitoring both rotational and lateral movements of biomolecules at the single-molecule level. Nevertheless, self-aggregation first needs to be supressed in order to utilize DNDs in intracellular environments. To do so, beads-assisted sonic disintegration, a combination of beads milling and strong sonication is predicted to be effective^40^. In addition, we succeeded in maintaining a mono-dispersed state in DND colloids by surface chemical modification using hyperbranched polyglycerol^41^.

In conclusion, the development of DND-OH, a single NVC-containing 5nm-sized label, will open the door for monitoring intracellular physical and chemical properties, including temperature, dynamics and interactions, of live cells and biomolecules at single-molecule resolution in an individual cell.

## Methods

### Sample preparation and characterization

A colloidal solution of DNDs (NanoAmando^®^, size 5.3 ± 1.0 nm; a kind donation from Dr. Eiji Osawa) was freeze-dried and thermally annealed at 800 °C for 2 h under vacuum to create NVCs, and then oxidized at 425 °C for 5 h to remove surface graphite (DND-bare). Surface OH termination on DNDs (DND-OH) was performed as described by Krüger *et al*.^21^. FTIR spectra were obtained on a JASCO FT/IR 4200 spectrometer using standard KBr pellets with a resolution of 2 cm^−1^. The zeta potentials of the samples in neutral water were measured by using a Malvern Zeta sizer Nano instrument. TEM images were obtained by using a JEOL JEM-1011 microscope. X-ray diffraction patterns were recorded by using a power X-ray diffractometer (Rigaku MultiFlex DR) with a CuKα source (X-ray wavelength 0.15405 nm) with an acceleration voltage and anode current of 30 kV and 30 mA, respectively.

### Fluorescence and ODMR measurements

The field of view was illuminated with an Nd:YAG laser (100 mW, 532 nm; Sapphire 532 LP, Coherent), and fluorescence images were obtained by an EMCCD camera (Andor iXon DU897, Andor Technology). We recorded fluorescence spectra by using a Hamamatsu photonics PMA-12. The emitted light was collected by an oil-immersion 60x or 100x (for super-resolution imaging) objective (CFI Apochromat TIRF 60x/1.49 and 100x/1.49, respectively; Nikon), passed through a dichroic mirror centred at 575 nm and a long-wave (short-wave) pass filter between 590 nm and 845 nm to detect the fluorescence signal from NVCs. The microscope was equipped with a microwave coil and a detachable neodymium magnet above the sample stage for irradiating the resonant frequency and external magnetic fields. Through this experimental setup, the ODMR intensity was calculated in accordance with equation (1), and the ODMR spectrum was obtained with the microwave frequency swept widely from 2670 to 3070 MHz point by point either with or without an external magnetic field. The ODMR spectrum in Fig. 2(a) and both pulse ODMR experiments in Fig. 2(d) were recorded on a confocal setup.

## Electronic supplementary material

Supplementary Information
