# Supplementary material for: Enrichment of ODMR-active nitrogen-vacancy centres in five-nanometre-sized detonation-synthesized nanodiamonds: Nanoprobes for temperature, angle and position
Source: Sci Rep. 2018 Apr 3;8:5463. doi: 10.1038/s41598-018-23635-5 (PMC5883028; doi:10.1038/s41598-018-23635-5)
Supplement: Supplementary file 1 — Supplementary Information [file 41598_2018_23635_MOESM1_ESM.pdf]

Supplementary Information

Enrichment of ODMR-active nitrogen-vacancy centres in five-nanometre-sized detonation-synthesized nanodiamonds: Nanoprobes for temperature, angle and position

Shingo Sotoma[1], Daiki Terada[1], Takuya F. Segawa[1,2], Ryuji Igarashi[1,3,*], Yoshie Harada[4,5,*] and Masahiro Shirakawa[1,5,*]

[1]Department of Molecular Engineering, Graduate School of Engineering, Kyoto University, Nishikyo-Ku, Kyoto 615-8510, Japan; [2]Laboratory for Solid State Physics, Eidgenössische Technische Hochschule (ETH) Zürich, CH-8093 Zürich, Switzerland; [3]PRESTO, Japan Science and Technology Agency, Kawaguchi 332-0012, Japan; [4]Institute for Protein Research, Osaka University, Yamadaoka, Suita, Osaka 565-0871, Japan; [5]Institute for Integrated Cell-Material Sciences (WPI-iCeMS), Kyoto University, Yoshida-Honmachi, Sakyo-ku, Kyoto 606-8501, Japan.

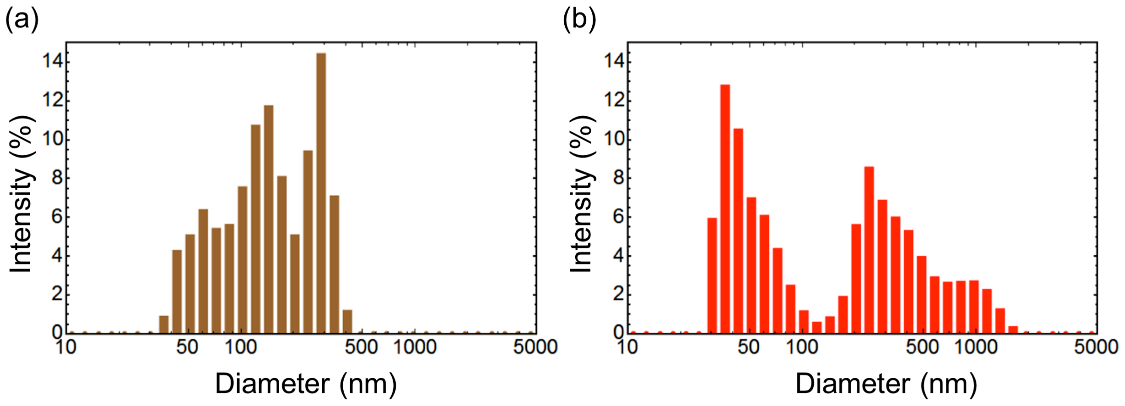

Figure S1. Particle size distribution by particle number of aggregates of (a) DND-bare (brown) and (b) DND-OH (red) determined by dynamic light scattering. Both DNDs form aggregation in water. The sizes were determined using a Nikkiso Microtrack II.

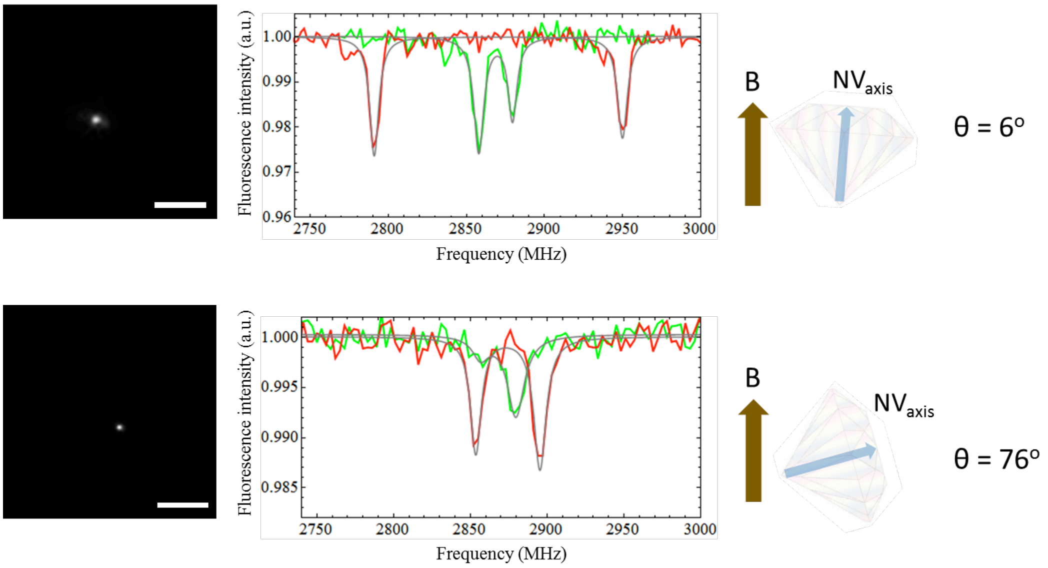

Figure S2. Two examples of a fluorescence image, ODMR spectrum and schematic of the NV axis' orientation toward the applied magnetic field, estimated from the corresponding ODMR spectrum. Red and green ODMR spectra denote the presence (red) and absence (green) of the 28-Gauss external magnetic field, respectively. The scale bar in the fluorescence image is 10 μm.

Error estimation of NV orientation relative to external magnetic fields.

To estimate the precision of the NV orientation relative to the external magnetic field from an ODMR signal, we performed the following Monte Carlo error simulation. First, we measured the ODMR spectrum under a 28-Gauss external magnetic field at each acquisition time of the spectrum and fitted the spectrum by two Lorentzian functions (Fig. S3a). The NV orientation was then determined from the resonant point in the ODMR spectrum using the spin Hamiltonian (Equation 1 in the main text). Next, we calculated the base noise level in the ODMR spectrum and its standard deviation (σ) (Fig. S3b). We hypothesized that the noise in the ODMR spectrum always distributes as a Gaussian with a standard deviation of σ. We then added a randomly drawn error to the fitted ODMR spectrum. After 1000 random (Monte Carlo) simulations, we obtained a distribution of resonant frequencies in the spectrum (Fig. S3c and d), which indicates the error in the NV orientation. This distribution was fitted to a Gaussian and its standard deviation was determined. From the results, we estimated the error in the orientation for each acquisition time of the spectrum.

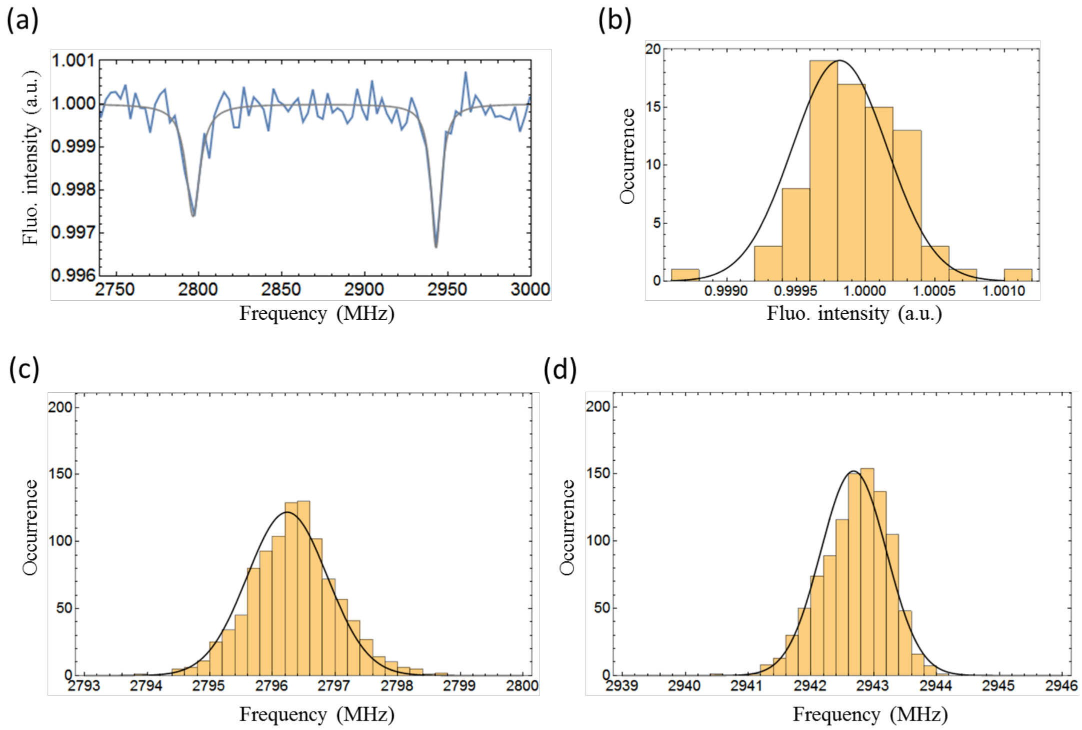

Figure S3. (a) Typical ODMR spectrum (gray) with an acquisition time of 500 ms per data point. The spectrum is fitted by two Lorentzian functions. (b) Distribution of fluorescence intensity at the base level of the ODMR spectrum. The distribution is fitted to a Gaussian, and its σ is estimated. (c) and (d) show distributions of the resonant point obtained in Monte Carlo simulations of ms = 0 to −1 and ms = 0 to +1, respectively. Data are again fitted by Gaussians.

ODMR-based single-emitter switching technique of DNDs

Point-localization based super-resolution microscopy techniques normally require single emitter switching. For instance, PALM and STORM can be performed by stochastic photoactivation of fluorophores. Using the ODMRs of nanodiamonds, we instead achieve reliable single-emitter switching by alternating the MW frequency under an appropriate magnetic field.

Fig. S4a shows a conventional fluorescence image of two DNDs (red and blue circles). The DNDs are outside the diffraction-limit proximity, thus distinguishable from each other. We measured the ODMR spectrum of the DNDs in the presence of an external magnetic field. The spectrum (Fig. S4b) exhibits two pairs of Zeeman-doublet ODMR signals (red and blue, corresponding to the red and blue circles in the fluorescence image, respectively). Therefore, each DND has distinct resonance frequencies (2820 and 2920 MHz and 2775 and 2955 MHz, respectively), indicating that their crystal lattices point in different directions. This feature can be used for single emitter switching. By irradiating microwaves at 2820 and 2775 MHz, the fluorescence emitted by NVCs were selectively corrected and then the NVC-selective images of DNDs were constructed (see Fig. S4c and d).

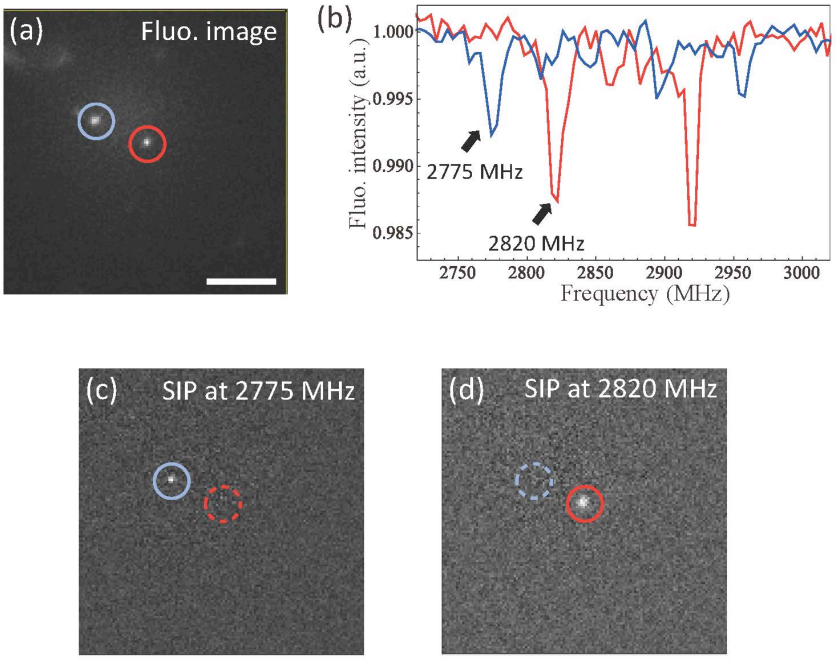

Figure S4. (a) Fluorescence images of two DNDs (scale bar is10 μm), each indicated by a blue or red circle. (b) ODMR spectra corresponding to these DNDs. (c) NVC-selective image under MW irradiation at 2775 MHz. The fluorescent spot in the red circle apparently disappears, while the spot in the blue circle is selectively extracted. Conversely, NVC-selective at 2820 MHz extracts the fluorescence in the red circle and diminishes the fluorescence in the blue circle.

Error in central position, inherent in the experimental set-up.

We estimated the inherent error in our experimental set-up conditions as follows. The obtained ODMR images (red or blue plots in Fig. 4c; see main text) were displayed as the integrated image data of 200 frames. The data were divided into odd and even frames and subsequently fitted by 2D Gaussians. The central positions of both frames were then determined. Ideally, the central positions of the odd and even frames should coincide. Fig. S5 presents average ODMR images of odd and even frames of DNDs (indicated as blue and red plots in Fig. 4c of the main text, respectively). We find that the central point differs between the odd and even frames. This difference was taken as the error in the experimental set-up conditions.

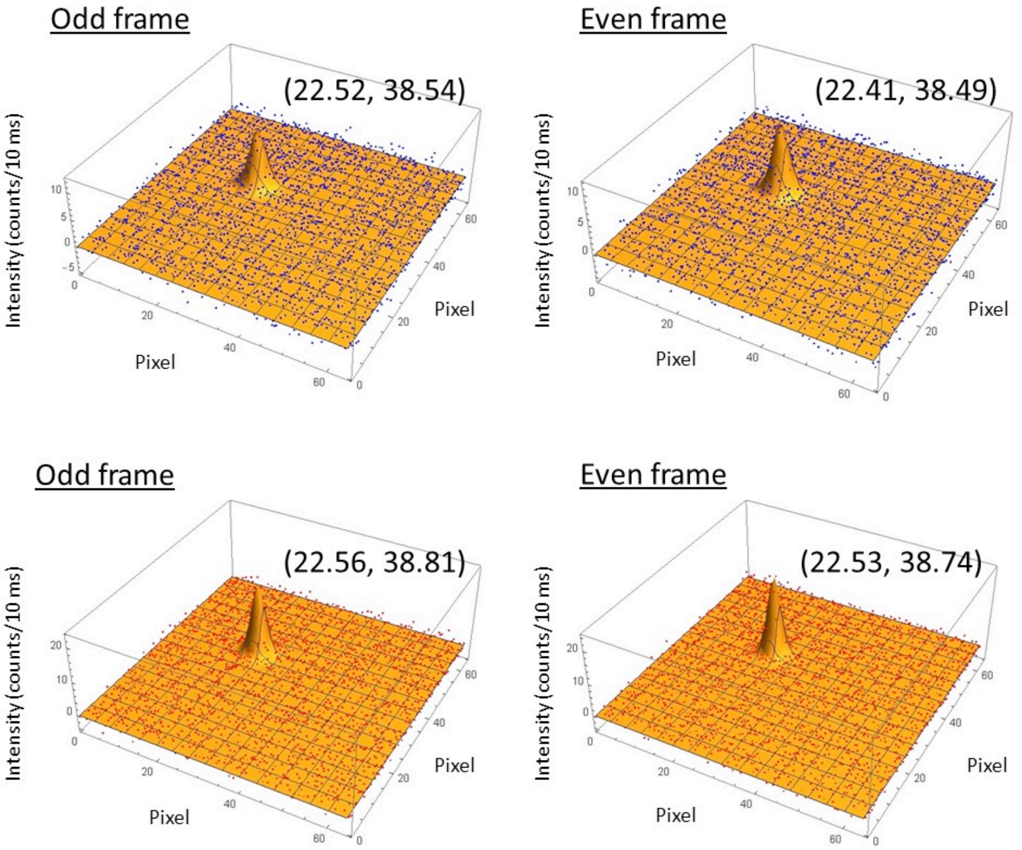

Figure S5. Odd and even frames of ODMR image of DNDs that is indicated by blue plot (upper two images) or red plot (lower two images) in Fig. 4c in the main text. Note that blue and red plots show the ODMR intensity with fixing MW at 2857 and 2821 MHz, respectively.
